# An evaluation for the medium-term storage and viability of root cortex tissues stained with blue ink in the assessment of arbuscular mycorrhizal fungi

**DOI:** 10.1099/acmi.0.000292

**Published:** 2021-11-30

**Authors:** Thomas Wilkes, Douglas Warner

**Affiliations:** ^1^​ Department of Psychology, Sport and Geography, School of Life and Medical Sciences, College Lane Campus, University of Hertfordshire, Hatfield, Hertfordshire AL10 9AB, UK; ^2^​ Agriculture and Environment Research Unit, School of Life and Medical Sciences, College Lane Campus, University of Hertfordshire, Hatfield, Hertfordshire AL10 9AB, UK

**Keywords:** arbuscular mycorrhizal fungi, blue ink stain, degradation, storage, low toxicity stain, winter wheat root

## Abstract

Sheaffer blue ink is an effective method to stain arbuscular mycorrhizal (AM) fungi in a variety of plant species. It has, however, received criticism for its potential rapid degradation and short-term viability. The long and medium term storage and viability of stained samples has not, to date, been described for this particular staining method. This short communication reports on the viability of 730 samples stained with Sheaffer blue ink stored for the duration of 4 years in microscope slide boxes out of direct sunlight. There was no significant difference in micrograph image quality and presence of stain between years as indicated by the number of AM fungal structures quantified. In conclusion Sheaffer blue ink stain does not deteriorate in the medium term.

## Impact Statement

The present communication evaluates the medium-term viability of Sheaffer blue ink staining for arbuscular mycorrhizal fungi. To date, Sheaffer blue ink stained root materials have not been subject to a further quantification and assessed for the longevity of the original stain during prolonged storage. The samples evaluated in the present communication show that root tissues retain the original stain and maintain the capacity to identify AM fungal structures for a minimum of 4 years.

## Introduction

Arbuscular mycorrhizal (AM) fungi are symbiotic biotrophs [1] that are thought to have aided in the initial land colonisation of terrestrial plants approximately 400 to 480 million years ago [2]. Of commercial importance, AM fungi are of economic benefit in agriculture for a number of reasons. Plant-mycorrhizal associations provide nutrient transfer from soil to plant via the peri arbuscular membrane [3] in exchange for photosynthetic carbohydrates accounting for an estimated 20% of total photosynthetic carbon produced by the host plant [4]. Furthermore, AM fungi have been attributed to the production of the glycoprotein glomalin, which has been correlated with increased soil aggregation and soil quality [5]. As such, their abundance and distribution within agricultural systems has been subject to analysis. Several methods have been proposed for the quantification of AM fungi, including intracellular root component staining [6].

Many current staining procedures utilise trypan blue (C_34_H_28_N_6_O_14_S_4_) or lactophenol cotton blue as a method of detecting mycorrhizal structures such as vesicles, arbuscules and intra-radiating hyphae [7]. Trypan blue stains AM fungal vesicles and spores in a similar manner, making distinguishing these difficult and risking inflated quantification of mycorrhizal associations. Sheaffer blue ink represents a non-carcinogenic alternative stain for mycorrhizal structures [7–11]. A review of staining protocols by Moukarzel *et al*. [12] criticises the use of Sheaffer blue ink due to its perceived deterioration and loss of clarity. The authors do not, however, appear to substantiate this experimentally themselves, rather they cite a study by Vierheilig *et al*. published in 2005 [13] following work originally conducted in 1998 [14]. No further evaluation of stained roots was made between these dates. As highlighted by Wilkes *et al*. [7], although Vierheilig *et al*. [13, 14] investigate multiple coloured inks as potential staining mediums, they do not provide additional data or clear photographic images to substantiate their conclusions. Further, the use of different coloured inks, for example black, by Veierheilg *et al*. [14] may not be as effective as the blue ink used by Wilkes *et al*. [7]. Another critical factor, seemingly overlooked by Moukarzel *et al*. [12], is that the protocol of Veierheilg *et al*. [14] cleans the roots with 10% w/v potassium hydroxide (KOH) as a method to remove debris. This approach reduces the structural integrity of the root cells by chemical degradation of the cell wall [15] most likely resulting in the deterioration observed by Veierheilg *et al*. [13]. Further to this, Wilkes [16] comment on the use of KOH during the process of ergosterol quantification as a proxy indicator of mycorrhizal fungal biomass. The authors observed that KOH damaged root cortical AM fungal structures while simultaneously elevating the quantity of measured ergosterol. The higher temperature associated with the boiling of roots in KOH in the protocol of Veierheilg *et al*. [14] results in further damage to fungal cell membranes. An increase in membrane damage risks a more rapid deterioration of staining. The Sheaffer blue ink approach used by Wilkes *et al*. [7] clears the roots with sonification as an alternative approach, avoiding the risk of cell wall degradation attributed to the application of 10% w/v KOH at high temperatures. Further, Veierheilg *et al*. [14] do not provide details of the conditions (light, temperature, humidity) under which the ink stained root samples were stored, a factor considered in this communication.

This communication aims to assess the longevity of wheat root sections stained with Sheaffer blue ink according to the protocol of Wilkes *et al*. [7]. The clarity of mycorrhizal structures in winter wheat root sections has been quantified annually for a period of 4 years after the samples were originally stained. The implications for the viability of Sheaffer blue ink as a stain coupled with sonification as a method of debris removal and the continued viability of stored samples are discussed.

## Methods

**Fig. 1.**
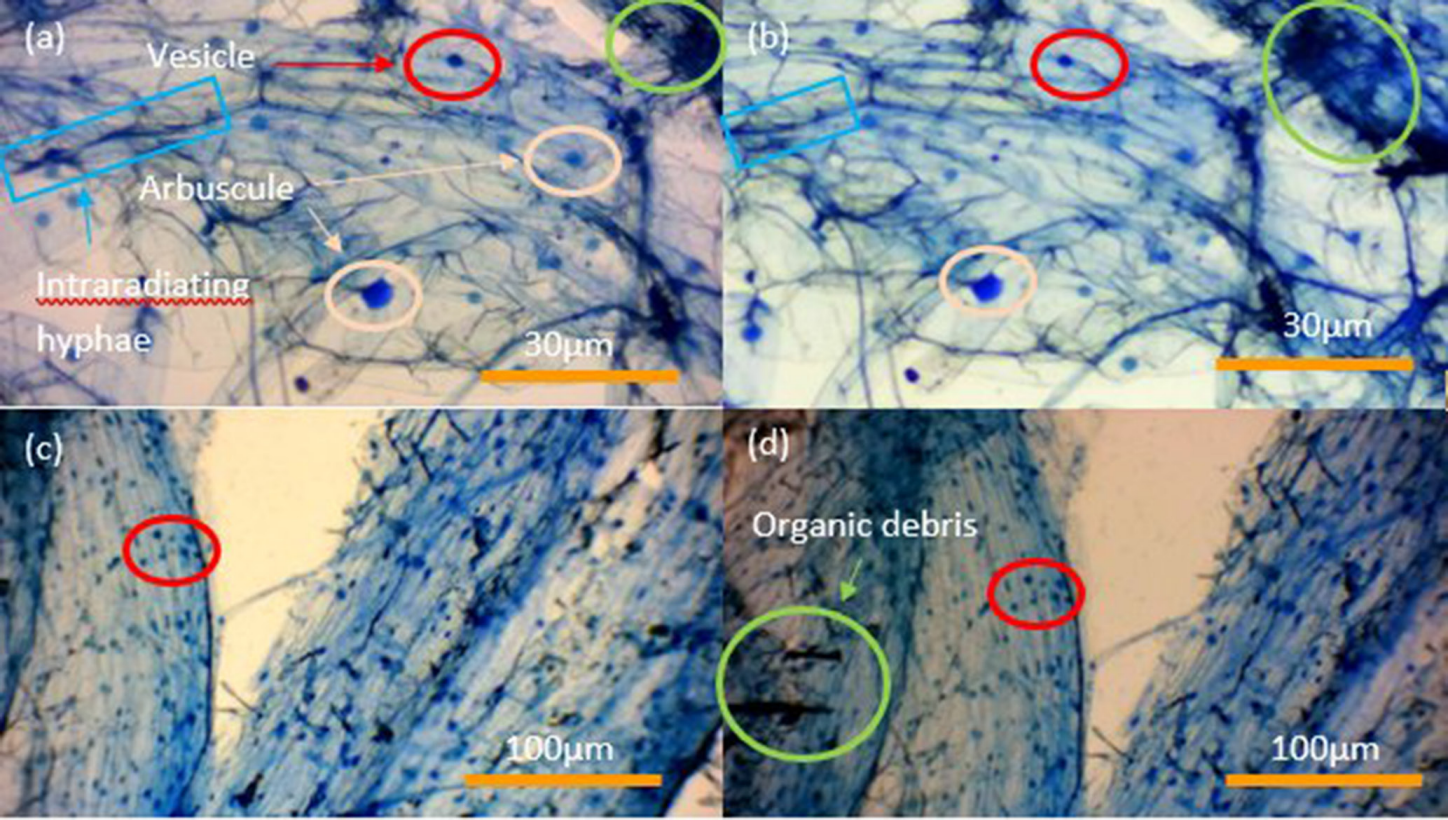
Micrographs of winter wheat taken in (**a**) February 2017, (**b**) March 2021, (**c**) October 2020, and (**d**) March 2021 using a Bresser HD microscope camera at their respective magnifications. Micrographs (**a**) and (**b**) are of the same stained root sections from their respective wheat sample. This is also true for micrographs (**c**) and (**d**). Red: vesicle, Blue: intraradiating hyphae, Peach: arbuscule, Green: organic debris.

**Fig. 2.**
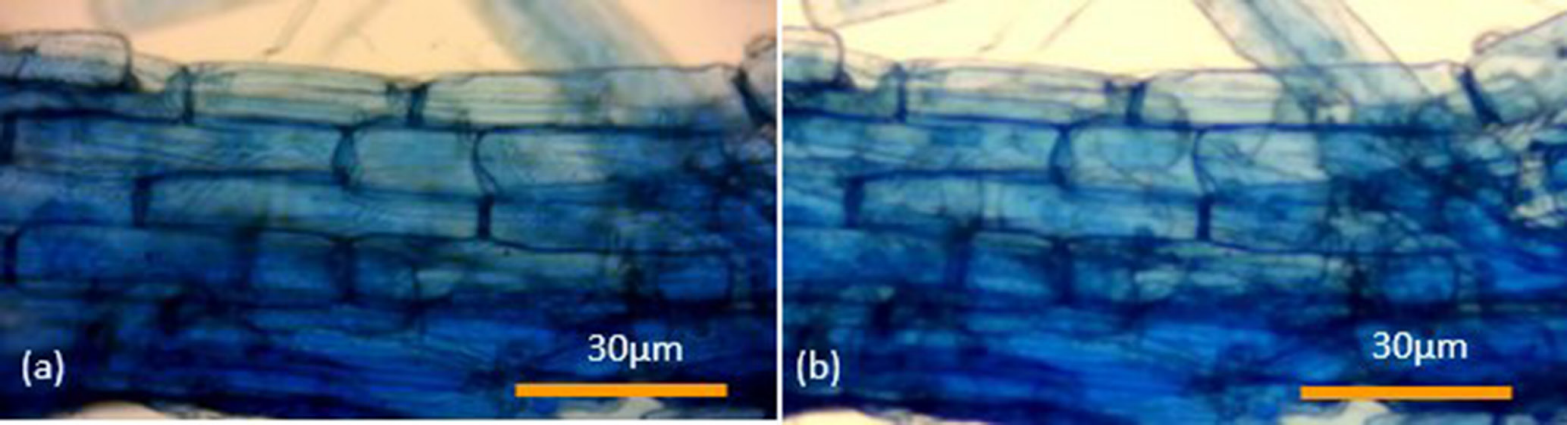
Micrographs of winter wheat taken in (**a**) February 2017 and (**b**) March 2021 using a Bresser HD microscope camera at ×100 magnification. Wheat was grown in media void of arbuscular mycorrhizal structures. Root staining confirmed the absence of arbuscular mycorrhizal structures. Micrographs (**a**) and (**b**) are of the same stained root section from their respective wheat sample.

Root cortical samples of winter wheat (variety Zulu) stained with Sheaffer blue ink (*n*=730) according to the protocol of Wilkes *et al*. [7] have been examined and intracellular root arbuscules and vesicles quantitated annually at periods of between one and 4 years after the root tissues were originally stained. Soil particles were removed from root systems by ultrasonic water bath (Bandelin Sonorex Super, Berlin, Germany) at 42 KHz for 10 min, without heating, then rinsed in deionised water. Samples were fixed in a 10% formaldehyde (CH_2_O), 50% ethyl alcohol (CH_3_CH_2_OH), 5% acetic acid (CH₃COOH) v/v (FAA) solution for 24 h, autoclaved in deionised water and incubated at 60 °C in 5% v/v hydrochloric acid (HCl) for 1 h. Roots were divided into 5×1 cm sections and stained with 10% v/v Sheaffer Blue ink in 5% glacial acetic acid for 3 min, subject to root squash with root tissues sealed between microscope slides with clear nail varnish (nitrocellulose [C_6_H_7_(NO_2_)_3_O_5_] dissolved in ethyl acetate [C_4_H_8_O_2_]) and viewed under a light microscope at ×100 magnification. Samples were stored at room temperature in microscope slide boxes avoiding direct sunlight. Images of samples (Figs. 1 and 2) were taken with a Bresser HD microscope camera.

## Statistical analysis

Statistical analyses were conducted using the R commander (Hamilton, ON, Canada) software package. Arbuscular mycorrhizal fungal root arbuscules were quantified both visually and using CamLabLite version 1.0.8942.20170412 (Bresser, Rhede, Germany). The mean and standard error was calculated for each set of sample data. A single factor ANOVA tested for differences in winter wheat root mycorrhizal structures between years before further statistical testing was performed. Paired two-tail *t*-tests of equal variance compared the baseline year (the year that the root samples were stained) with observations of root arbuscules for the same samples in the year 2021 (between one and 4 years after initial staining). Statistical significance was determined by *P* values ≤0.05.

## Results

**Fig. 3.**
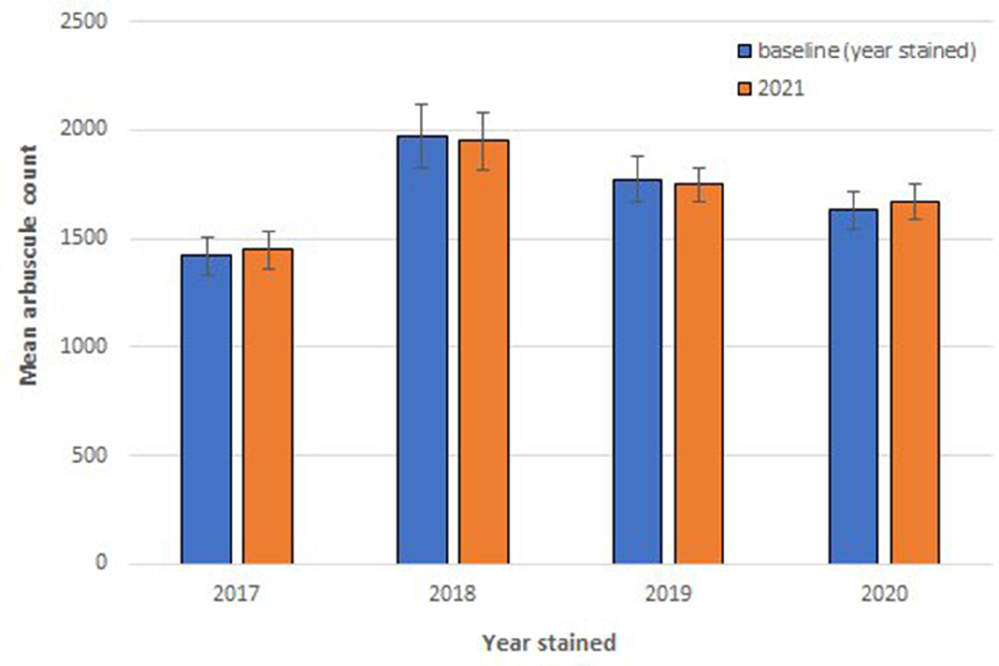
Mean arbuscule count per 1 cm root section from Sheaffer blue stained wheat quantified in 2021 from root tissues originally stained in years 2017 (*n*=80), 2018 (*n*=150), 2019 (*n*=350), and 2020 (*n*=150). The number of arbuscular mycorrhizal (AM) fungal arbuscules in 2021 were compared with the previous number for their respective years of staining and the mean difference calculated. Error bars were constructed from standard error of the mean (SEM).

Single factor ANOVA testing revealed no statistical difference between AM fungal root structures between the years quantified (Fig. 3) (*P*=0.76, df: 4, 256, F value: 51.90, F critical: 2.64, Single factor ANOVA). A paired two-tail *t*-test of equal variance revealed no significant difference (Fig. 3) between the number of quantified winter wheat root AM fungal structures originally and observed again in years one to four (*P*=0.95, df: 345, t.stat: 0.07, paired equal variance T test).

## Discussion

The present communication evaluates the medium-term viability of Sheaffer blue ink staining for AM fungi structures in winter wheat roots when stored under appropriate conditions. It contests the short-term viability of Sheaffer blue ink staining reported by Vierheilig *et al*. [13] and cited by Moukarzel *et al*. [12].

Modifications to the ink staining approach, implemented by Wilkes *et al*. [7], who use blue ink and sonification instead of black ink and 10% w/v KOH at boiling point as a method of debris removal, appear to mitigate the deterioration of stained samples compared to the findings reported by Vierheilig *et al*. [13]. Recent studies utilising Sheaffer blue ink [7–11] identify increased clarity of stained root tissues relative to trypan blue coupled with reduced soil and organic debris through sonification as an alternative to the 10 % w/v KOH at temperature above 60 °C previously used by Vierheilig *et al*. [14]. Additionally, as discussed by Wilkes *et al*. [7] staining mycorrhizal fungi with black ink does not allow for the differentiation between fungal structures, fungal spores and organic debris remaining on root systems.

Vierheilig *et al*. [13, 14] did not employ a fixative solution, such as the formaldehyde, alcohol, acetic acid solution (FAA) described by Wilkes *et al*. [7–9]. Kowel *et al*. [10, 11] also use a fixative solution, however they removed the formaldehyde component of the FAA solution to fix plant root tissues. The micrographs presented by Kowel *et al*. [10, 11] demonstrate a negligible difference in the stain quality and clarity of the AM fungi image compared to those produced by Wilkes *et al*. [7–9] The impact of the removal of the formaldehyde component of the root fixative prior to Sheaffer blue staining on the medium-term storage capability of stained root tissues is unknown. Moukarzel *et al*. [12] fail to take into consideration the difference in the methodologies of Kowel *et al*. [10, 11] and Wilkes *et al*. [7–9] to those of Vierheilig *et al*. [13, 14] when commenting that Sheaffer blue ink stain is unsuitable for storage in the medium to long term.

## Conclusion

The present communication has been able to demonstrate that Sheaffer blue ink, in the staining of AM fungal root components, has medium term storage potential when root tissues are fixed in FAA solution. The avoidance of boiling in KOH and using a fixative afterwards preserves AM structures, improves staining clarity and longevity. The continued monitoring of sample clarity and an evaluation of the impact of storage procedure, for example the effects of temperature and sunlight, on the persistence of ink as a mycorrhizal stain will be undertaken as future work.
